# Quantum Secure Multi-Party Summation with Graph State

**DOI:** 10.3390/e26010080

**Published:** 2024-01-17

**Authors:** Yaohua Lu, Gangyi Ding

**Affiliations:** School of Computer Science and Technology, Beijing Institute of Technology, Beijing 100081, China; dgy@bit.edu.cn

**Keywords:** quantum cryptography, quantum secure multi-party summation, quantum graph state

## Abstract

Quantum secure multi-party summation (QSMS) is a fundamental problem in quantum secure multi-party computation (QSMC), wherein multiple parties compute the sum of their data without revealing them. This paper proposes a novel QSMS protocol based on graph state, which offers enhanced security, usability, and flexibility compared to existing methods. The protocol leverages the structural advantages of graph state and employs random graph state structures and random encryption gate operations to provide stronger security. Additionally, the stabilizer of the graph state is utilized to detect eavesdroppers and channel noise without the need for decoy bits. The protocol allows for the arbitrary addition and deletion of participants, enabling greater flexibility. Experimental verification is conducted to demonstrate the security, effectiveness, and practicality of the proposed protocols. The correctness and security of the protocols are formally proven. The QSMS method based on graph state introduces new opportunities for QSMC. It highlights the potential of leveraging quantum graph state technology to securely and efficiently solve various multi-party computation problems.

## 1. Introduction

As cloud computing and big data become more prevalent, data security and privacy protection are increasingly important. Secure multi-party computation is a mode of computation that protects the privacy of inputs, and it has a wide range of applications in fields such as e-commerce, medical service, financial transactions, etc. Its main goal is to enable two or more parties to perform a confidential computation task without revealing their own inputs to each other or to anyone else. Yao et al. [1] proposed the millionaire problem in 1982, which was the first to introduce the idea of secure computation. Since then, various secure multi-party computation problems have been proposed and solved, such as privacy comparison [2,3,4,5], secure summation [6,7,8], set intersection and union [9,10], Manhattan distance [11], and others. Secure multi-party computation in the classical domain relies on classical homomorphic encryption techniques based on hard mathematical problems. However, with the development of quantum computing technology, classical secure multi-party computation faces the threat of quantum computing. Shor’s algorithm [12] challenges the hard mathematical problems in the classical domain. Quantum secure multi-party computation, on the other hand, is based on the principles of quantum mechanics, providing superior security performance and the ability to easily detect eavesdroppers. The BB84 quantum key distribution protocol [13] initiated the research on quantum cryptography. Since then, many QKD protocols have been proposed and experimentally demonstrated, aiming to overcome some practical challenges, such as the rate-loss limit, the finite-key effect, and the coherent attack. In 2018, Lucamarini et al. [14] proposed and demonstrated a QKD protocol that overcomes the rate-loss limit without quantum repeaters, using a technique called twin-field QKD. In 2021, Proietti et al. [15] realized an experimental quantum conference key agreement among eight parties, using a quantum network based on entangled photon pairs. In 2022, Zeng et al. [16] proposed and experimentally verified a QKD protocol that uses mode-pairing to achieve high key rates and robustness against channel noise. Gao et al. [17] presented a simple security proof of coherent-one-way QKD, which is a QKD protocol that uses weak coherent pulses and one-way post-processing. Lavie et al. [18] improved the coherent-one-way QKD protocol for high-loss channels, by introducing advantage distillation and decoy states. In 2023, Wang et al. [19] performed a tight finite-key analysis for mode-pairing QKD, which reduces the key consumption and improves the key rate. Yin et al. [20] demonstrated a quantum secure network with digital signatures and encryption. Zhou et al. [21] achieved experimental quantum communication that overcomes the rate-loss limit without global phase tracking. Schiansky et al. [22] implemented a quantum–digital payment system that combines QKD, quantum money, and blockchain technology. Furthermore, more and more researchers have applied quantum techniques to secure multi-party computation fields such as privacy comparison [23,24,25], secure multi-party summation [26,27,28], set intersection and union [29,30,31], Manhattan distance [32], etc.

In this paper, we address the quantum secure multi-party summation (QSMS) problem, a fundamental and important problem in quantum secure multi-party computation (QSMC). QSMS enables multiple parties to compute the sum of their input data in a privacy-preserving way. It serves as a building block for more complex secure multi-party computations. Let $P_{1},P_{2},...,P_{n}$ be the participants, and $x_{1},x_{2},...,x_{n}$ be their private data. The encryption function is f(x) and the decryption function is d(y). The goal is to securely compute the following equationtext:

(1) $$d\left(f\left(x_{1},x_{2},\ldots x_{n}\right)\right)=d\left(\sum_{i=1}^{n}f\left(x_{i}\right)\right)=x_{1}+x_{2}+\ldots +x_{n}$$

In 2002, Heinrich [33] proposed the idea of quantum summation, and collaborated with Kwas et al. [34] in 2004 to study the problem of quantum Boolean summation. In 2007, Vaccaro et al. [35] applied quantum summation to anonymous voting protocols. Since then, more and more researchers have started to study the problem of secure multi-party summation, and they have constructed protocols with unique features based on different quantum resources. In 2010, Chen et al. [26] proposed quantum two-party and multi-party secure summation based on GHZ states. In 2014, Zhang et al. [27] realized high-capacity quantum summation using single photons in polarization and spatial modes. In 2015, Zhang et al. [36] proposed a secure three-party summation protocol without the help of a semi-honest third party. In 2016, Shi et al. [28] constructed secure multi-party summation and multiplication protocols based on quantum Fourier transform. In 2017, Liu et al. [37] proposed a quantum secure multi-party summation protocol based on two-particle Bell states. In 2018, Yang et al. [38] proposed a tree-structured quantum secure multi-party summation protocol. In 2019, Ji et al. [39] proposed a quantum secure multi-party summation protocol based on entanglement swapping. In 2021, Zhang et al. [40] implemented a secure three-party semi-quantum summation protocol using single photons. In recent years, there have been some new research points on quantum secure multi-party summation protocols. In 2022, Ye et al. [41] proposed a semi-quantum summation scheme that is immune to collective dephasing noise and has stronger robustness. In the same year, Shi et al. [42] proposed device-independent secure multi-party modulo 2 summation and modulo d summation protocols based on MDI-QKD technology, using weak coherent pulses as quantum resources, and applying only simple-gate operations and Bell measurements, which have stronger practicality; Hayashi et al. [43] proposed a quantum secure multi-party summation protocol based on secure modulo zero-sum randomness; Cai et al. [44] pointed out that traditional secure summation protocols may suffer from impersonation attacks wherein fake data are sent by impostor parties, resulting in erroneous summation results, and proposed an improved scheme. In 2023, Wang et al. [45] proposed an identity authentication method based on exchange encryption, which can solve the problem of impersonating parties in secure multi-party summation protocols. In the same year, Li et al. [46] proposed a new quantum secure multi-party summation protocol based on Shamir’s threshold scheme and d-dimensional GHZ states, which uses a (k,n)-threshold method, and has a lower computational cost than the (n,n) threshold quantum secure multi-party summation protocol.

Generally speaking, researchers have conducted in-depth studies on quantum secure multi-party summation protocols using different quantum resources. However, most of these protocols are based on the quantum gate circuit model, where classical information is encoded into quantum states by fixed quantum state preparation or fixed gate operations, and the security of quantum channels is ensured by adding and measuring decoy bits; thus, the security of the information needs to be improved further. Quantum graph states, as a kind of quantum states that describe the complex entanglement relationship of multi-body quantum systems, have advantages such as scalability, measurement friendliness, and error tolerance, provide a stronger security than the methods based on the quantum mechanics itself by using random graph state structures and random encryption gate operations, which provide a new idea for quantum secure multi-party computation. But from the current point of view, quantum graph states are mainly used as a technical framework for the implementation of quantum algorithms, and the research on applying quantum graph state technology to quantum secure multi-party summation is still relatively rare. Raussendorf [47] first proposed the concept of quantum Cluster states in 2001. Based on this, Hein et al. [48] proposed multi-body entanglement based on quantum graph states in 2004. In 2016, Liang et al. [49] proposed a quantum secret sharing protocol based on quantum graph states, applying the idea of matrix splitting method to the quantum domain. In 2019, Tian et al. [50] proposed a multi-party collaborative quantum computation protocol based on redundant graph states, using a special graph state structure to achieve multi-party collaborative computation. In 2020, Dou et al. [51] proposed protocols such as privacy comparison and multi-party secure summation based on quantum graph states, using some basic measurement properties of graph states to achieve secure multi-party summation. However, these studies only apply fixed graph state structures and fixed gate operations.

In this paper, we propose a graph state-based secure multi-party summation protocol based on previous research. First, each participant prepares a random graph state structure to hold data, encrypts data with a private key, and encodes data with random gate operations. Second, the participants send the graph state to a semi-honest third party (TP) and announce their graph state structure. We assume that the TP will follow the protocol honestly, but may try to learn additional information from the messages he receives or sends. The TP performs stabilizer measurements according to the graph state structure announced by the participants. If any stabilizer is in the −1 eigenstate, this indicates that eavesdropping or channel noise may have occurred during the transmission, the graph state structure is destroyed, and the protocol is terminated; if all stabilizers are in the +1 eigenstate, this indicates that the graph state has been securely transmitted. The TP then performs measurements according to the announced graph state structure and recovers the data bits (encrypted by the private key). Finally, after obtaining all the data, the TP performs summation and sends the summation data to the participants for joint decryption. Since the TP does not need to send quantum resources to the participants in advance, the number of participants can change at any time. The TP only needs to compute those graph states that have undergone secure transmission; thus, the protocol has higher flexibility. This paper designs a graph-based secure two-party sum protocol and two secure multi-party sum protocols (tree-shaped and ring-shaped) to adapt to different application scenarios. These protocols provide a new idea for applying graph state technology to solve secure multi-party computation problems and lay a foundation for extending other secure multi-party computation problems in the future.

The contributions of this paper are as follows:

1. We propose a secure multi-party summation protocol based on graph states, capable of solving both secure two-party summation and secure multi-party summation problems.

2. We apply the properties of graph states, such as structural security, scalability, and measurement friendliness, to solve secure multi-party computation problems, offering a novel approach to the application of graph states.

3. The protocol utilizes random graph state structures and random encryption gate operations, enhancing security and efficiency compared to previous protocols. It also allows for the dynamic addition and deletion of participants, increasing flexibility.

4. Experimental verification is also conducted to showcase its effectiveness and practicality. We prove the correctness and security analyses of the protocol. We provide detailed explanations of the application methods of various graph state properties.

**Figure 1:**
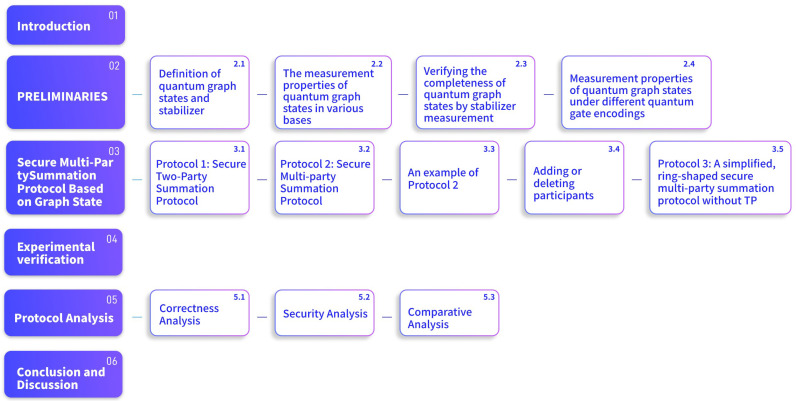
The structure of the paper.

The structure of this paper is shown in Figure 1. Section 2 introduces the basic properties of quantum graph states. Section 3 presents the specific content of the quantum secure two-party summation protocol and secure multi-party summation protocol. Section 4 verifies the effectiveness and practicality of the protocol through experiments. Section 5 proves the correctness and security of the protocol, and provides a comparative analysis of the protocol. Finally, Section 6 provides a summary and outlook.

## 2. Preliminaries

This section presents the fundamental notions and distinctive features of quantum graph states.

### 2.1. Definition of Quantum Graph States and Stabilizer

Quantum graph states are composed of many vertices and edges. Vertices represent quantum bits, and edges represent the entanglement relationship between quantum bits. G = (V,E) denotes a graph, where V represents the set of vertices, and E represents the set of edges. For any vertex a∈V, and its adjacent vertex b∈V, there is {a,b}∈E. The process of generating a graph state is as follows:

1. Apply H gate to all vertices, resulting in |+〉 state;

2. Apply CZ gate to all edges, such as $CZ_{ab}$, to make the basis entangled.

This generates the graph state |G〉.

(2) $$|G\rangle =\left(\prod_{\{a,b\}\in E}CZ_{ab}\right){|+\rangle }^{⊗V}$$

Next, we introduce the stabilizer representation of graph states. Stabilizers are very helpful for understanding graph states, because they can not only describe the structure of graph states, but also verify and correct them. For each vertex a∈V, let N(a) be the set of vertices adjacent to it. Vertex a applies X gate to itself, and applies Z gate to the vertices in N(a), which forms a stabilizer for vertex a. Each vertex has a stabilizer, and all stabilizers can fix a graph state. For a graph G, its stabilizer is denoted as $S\left(G\right)=\left\{S_{a}|a\in V\right\}$, where $S_{a}=X_{a}\prod_{b\in N(a)}Z_{b}$. When a stabilizer is applied to a graph state, the graph state remains unchanged, that is, $S_{a}|G\rangle =|G\rangle$.

Here are some examples of graph states and stabilizers.

**Figure 2:**
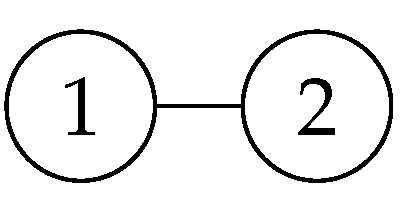
The linear graph state of two vertices connected by an edge.

It is the simplest graph state shown in Figure 2, generated by applying the H gate and the CZ gate to |00〉. $H_{1}H_{2}|00\rangle =|++\rangle .$ $CZ_{12}|++\rangle =\frac{1}{\sqrt{2}}\left(|0\rangle |+\rangle +|1\rangle |-\rangle \right)$. From this expression, we can see that applying $X_{1}Z_{2}$ to it results in $\frac{1}{\sqrt{2}}\left(|1\rangle |-\rangle +|0\rangle |+\rangle \right)$, which is obviously equal to the original expression, so its stabilizer is $X_{1}Z_{2}$. Similarly, the original expression can also be expanded as $\frac{1}{\sqrt{2}}\left(|+\rangle |0\rangle +|-\rangle |1\rangle \right)$. Obviously, $Z_{1}X_{2}$ is also its stabilizer. Expanding the expression further, the final result is $\frac{1}{2}\left(|00\rangle +|01\rangle +|10\rangle -|11\rangle \right)$, and its stabilizers are $X_{1}Z_{2}$ and $X_{1}Z_{2}$.

**Figure 3:**
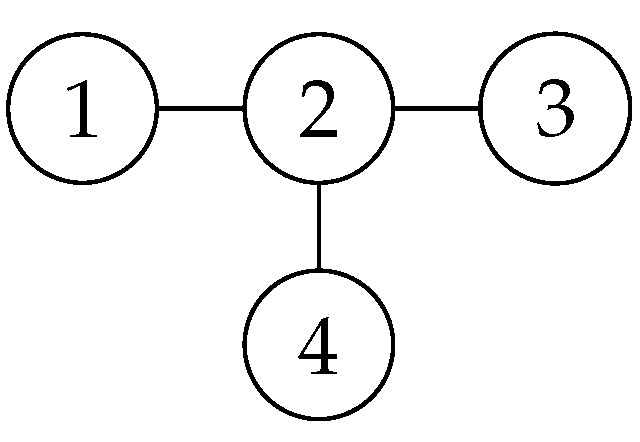
The four-vertex 2D square graph state.

The star graph state is shown in Figure 3. It is expressed in Dirac notation as: $\frac{1}{4}\left(|0000\rangle +|0001\rangle +|0010\rangle +|0011\rangle +|0100\rangle -|0101\rangle -|0110\rangle +|0111\rangle +|1000\rangle +|1001\rangle +|1010\rangle +|1011\rangle -|1100\rangle +|1101\rangle +|1110\rangle -|1111\rangle \right)$. The stabilizers are shown in Table 1.

These are the main graph state structures that are used in this paper, and other forms of graph states are similar.

### 2.2. The Measurement Properties of Quantum Graph States in Various Bases

**Figure 4:**
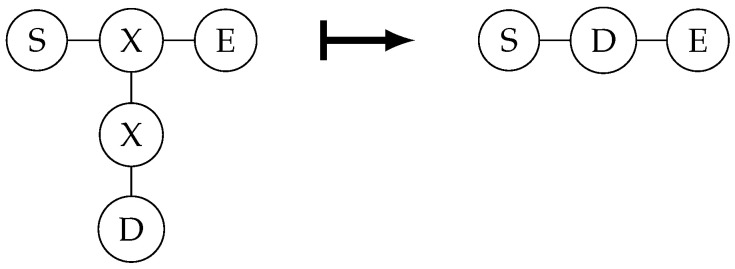
How the graph state changes after X-basis measurement.

The text below explains the measurement characteristics of quantum graph states under the X and Y bases. The first is the measurement on the X basis. The properties are shown in Figure 4.

It is easy to see that, when both X-basis measurement results are 0, it directly becomes the graph state on the right in the figure above. When the measurement result of the vertex above is 1, the Z gate needs to be applied to D; when the measurement result of the vertex below is 1, the Z gate needs to be applied to S and E.

**Figure 5:**
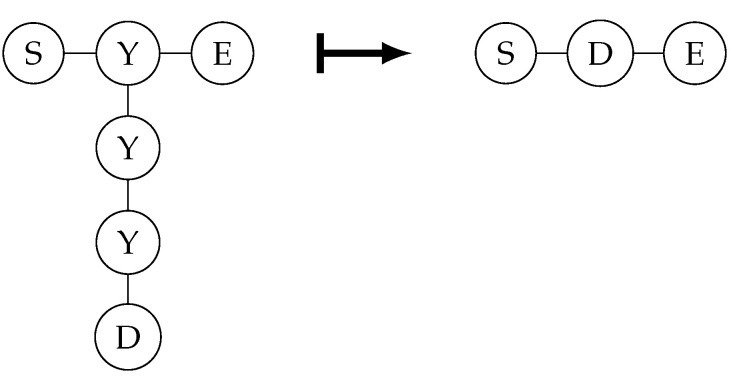
How the graph state changes after Y-basis measurement.

Next, consider the graph state measured in the Y-basis, whose properties are shown in Figure 5.

It can be easily derived that, when all three Y-basis measurement results are 0, it directly becomes the graph state on the right in the figure above. In other cases, some operations are needed. The truth table is shown in Table 2.

### 2.3. Verifying the Completeness of Quantum Graph States by Stabilizer Measurement

The important role of stabilizer coding is to verify the completeness of the graph state structure. In fact, all stabilizers constitute a set of commutative mechanical complete sets for the N-qubit system [52], so they have a set of common eigenstates. The graph state is the common eigenstate of all its stabilizer eigenvalues being +1. If the graph state structure changes, bit flips (X) or phase flips (Z) occur. For some stabilizers, its commutative structure was destroyed, and the eigenvalues of the stabilizers associated with the erroneous bits became −1. This is the basic principle that stabilizers can verify the completeness of graph states. Stabilizer measurement is an experimental method to implement the completeness detection of graph states, and the following steps are included: (1) Set an auxiliary bit c, initially |0〉, to test the eigenstate of a certain stabilizer. (2) For a certain stabilizer $S_{a}=X_{a}\prod_{b\in N(a)}Z_{b}$, apply the H gate, CNOT(a,c) gate, and H gate to the bit a, and apply the CNOT(b,c) gate to each bit b connected to a. (3) Measure the auxiliary bit c. If the result is 1, it means that the stabilizer is in the −1 eigenstate, the graph state structure is destroyed, or a flip occurs. It is easy to prove that, due to the special structure of the graph state, measuring the auxiliary bit c will not cause the collapse of the graph state. Note: If the measurement result of the auxiliary bit c is 0, it does not mean that the stabilizer must be in the +1 eigenstate, and some operations may cause the stabilizer to be in a superposition state. But if after multiple measurements all the stabilizer measurement results of the graph state are 0, the probability of graph state completeness will be very high.

### 2.4. Measurement Properties of Quantum Graph States under Different Quantum Gate Encodings

Finally, we discuss the changes in the graph state when X or Z gates are applied to encode data. First, we define the inverse operation of the graph state, which is performed to measure the encoded data. For a graph state $|G\rangle =\left(\prod_{\{a,b\}\in E}CZ_{ab}\right){|+\rangle }^{⊗V}$, we apply CZ gates to all connected edges, and then apply H gates to all vertices, to achieve the inverse operation of the graph state, $|G^{\prime }\rangle ={|+\rangle }^{⊗V}\left(\prod_{\{a,b\}\in E}CZ_{ab}\right)|G\rangle$. If the graph state is not encoded, we have $|G^{\prime }\rangle ={|0\rangle }^{⊗V}$. Next, we look at the measurement properties of the graph state for two vertices when X or Z gates are applied. For the simplest graph state, refer to Figure 2. First, we consider applying X gate to encode data on vertex 1 and Z gate to encrypt data on vertex 2. It is easy to find that, when the data are 00 or 11, according to the property of the graph state stabilizer, the graph state remains unchanged. Perform the inverse operation on the graph state. The result is |00〉. When the data are 10, apply the X gate to vertex 1, and the measurement result is |01〉. When the data are 01, apply the Z gate to vertex 2, and the measurement result is |01〉. It can be seen that, for this encoding method, the modulo 2 addition of the measurement results is the same as the modulo 2 addition of the original data. Using the same method, we can derive that, for two vertices using ZX gate, XX gate, ZZ gate to encode data can result in the same conclusion. That is to say, for the graph state of two vertices, randomly select X gate or Z gate to encode data, the measurement result of the graph state is the same as the modulo 2 addition of the original data. Moreover, if you do not know what kind of encryption gate operation is used on the two vertices, it is impossible to determine whether the data of the two vertices are 0 or 1 by the measurement result. This provides an idea for the privacy comparison and secure summation of the two participants.

**Figure 6:**
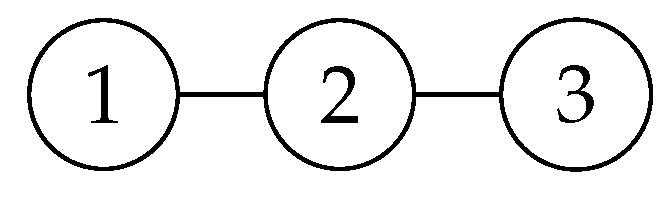
The three-vertex linear graph state.

To generalize the graph state situation, we examine the measurement outcomes of applying Z or X gates to a graph state with multiple vertices (e.g., three). Figure 6 shows an example of such a graph state.

When we apply the Z gate to encode data on vertex 2, the original graph state becomes $\frac{1}{2\sqrt{2}}\left(|000\rangle +|001\rangle -|010\rangle +|011\rangle +|100\rangle +|101\rangle +|110\rangle -|111\rangle \right)$. By applying the inverse operation of the graph state, the graph state becomes |010〉. The measurement result is consistent with the encoded data. When we apply the X gate to encode data on vertex 2, the original graph state becomes $\frac{1}{2\sqrt{2}}\left(|010\rangle +|011\rangle +|000\rangle -|001\rangle +|110\rangle +|111\rangle -|100\rangle +|101\rangle \right)$. By applying the inverse operation of the graph state, the graph state becomes |101〉, that is, three bits are flipped based on the original encoded data, and the measurement result can be obtained by flipping three bits. For |101〉, by taking the second bit as the center, the 123-bit flip is realized, and the original data can be obtained at |010〉. It is easy to derive that, for multiple qubits, randomly using X gate or Z gate to encode data, the Z-bit encryption part does not need to be decoded, and the X-bit encryption part is applied with bit flip, and the original data can be obtained. Random encryption gate operations further enhance the security of the data.

Furthermore, if we apply the X gate and Z gate to encode data on vertex 2, the original graph state becomes $\frac{1}{2\sqrt{2}}\left(|000\rangle -|001\rangle -|010\rangle -|011\rangle -|100\rangle +|101\rangle -|110\rangle -|111\rangle \right)$. By applying the inverse operation of the graph state, the graph state becomes |111〉; similarly, the result of applying Z gate and X gate encoding is −|111〉. The phenomenon of 3-bit flip after applying the X gate is the same as before. For the measurement result decoding, it can be realized by flipping three bits. For |111〉 and −|111〉, after decoding, they become |000〉 and −|000〉, which are consistent with the target results that X gate and Z gate (or Z gate and X gate) want to achieve. If a series of X gates and Z gates are applied to the same bit, because $X_{2}=I$, $Z_{2}=I$, and XZ=−ZX, it is known that it is the same as the modulo 2 addition result of the encoded data. That is to say, the data encoded by random X gate or Z gate are modulo 2 addition homomorphic, and the modulo 2 addition result of the original data can be obtained after decryption. It is also easy to find that, if multiple random gate operations are encrypted, the modulo 2 addition of the measurement results after decryption is the same as the modulo 2 addition of the original data.

That is to say, whether it is to measure after encoding the same graph state multiple times (equivalent to achieving summation during encoding), or to sum up after measuring multiple graph states separately, the same result can be obtained. This provides an idea for the secure multi-party summation of multiple participants. The specific proof will be introduced in the following sections.

## 3. Secure Multi-Party Summation Protocol Based on Graph State

This section presents a comprehensive overview of secure summation protocols, including a secure two-party summation protocol and two secure multi-party summation protocols based on graph state. The focus of this section is Protocol 2. We will give a specific example to illustrate how Protocol 2 is implemented, and explain the method of adding and deleting participants. Protocol 1 and Protocol 3 are simplified versions of Protocol 2.

### 3.1. Protocol 1: Secure Two-Party Summation Protocol

Protocol description: Alice and Bob encrypting and summing their respective data with the assistance of a third party (TP). The TP only knows the final summation result, but does not know the specific values of Alice and Bob. It should be noted that, if the TP announces the summation result, Alice and Bob can subtract the summation result from their own data, and thus infer the data of the other party. Therefore, achieving absolute security in a two-party summation protocol is not possible. However, in certain application scenarios, it may be feasible to enforce confidentiality by prohibiting the TP from disclosing the summation result. For instance, in a large-scale project bidding process, where the TP acts as the project initiator, Alice and Bob may collaborate to submit a joint bid. Both parties aim to maximize their individual amounts to maximize profits, while also striving for a competitive joint bid price. Prior to the announcement of the bid evaluation result, the TP is aware of the sum of Alice and Bob’s bids for comparison with other consortia, but the specific values of each party’s bid remain unknown to all three parties involved.

**Figure 7:**
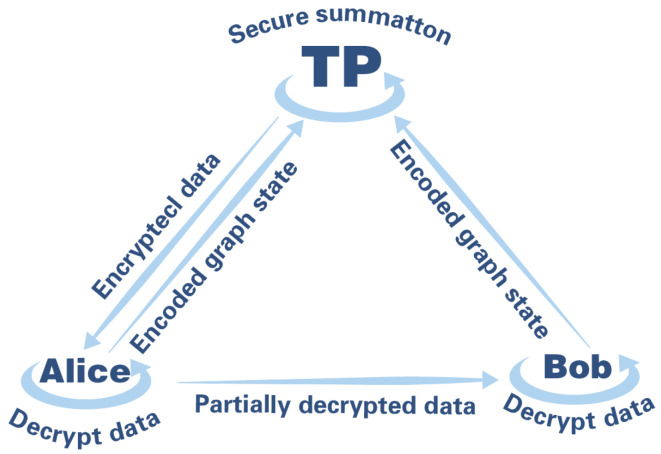
The process of two-party summation.

The specific secure two-party summation protocol is shown in Figure 7.

**Figure 8:**
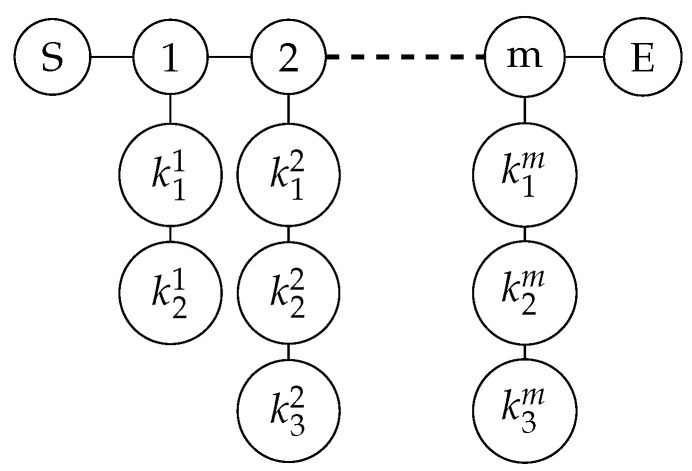
Prepare graph state according to Y.

Let Alice’s data be A = {$a_{i};i=1,2,\ldots \ldots ,m;a_{i}\in \left\{0,1\right\}$}, Bob’s data be B = {$b_{i};$$i=1,2,\ldots \ldots ,m;b_{i}\in \left\{0,1\right\}$}, m is a value far greater than the number of bits of both parties’ data, which is negotiated by both parties. The protocol goal is for TP to obtain f(A,B)=A⊕B. The specific steps of the protocol are as follows:

**Step 1: Prepare graph state.** Participants Alice and Bob, respectively, prepare private keys $Y_{A}=\left\{y_{i}^{A};i=1,2,\ldots \ldots ,m;y_{i}^{A}\in \left\{0,1\right\}\right\}$ and $Y_{B}=\left\{y_{i}^{B};i=1,2,\ldots \ldots ,m;y_{i}^{B}\in \left\{0,1\right\}\right\}$. According to the values of Y, participants Alice and Bob, respectively, prepare graph states, and each group of graph states includes S (start) bit and E (end) bit. If $y_{i}^{\left(AorB\right)}=0$, there are three vertices on each column, which are $k_{1}^{i}$, $k_{2}^{i}$, and $k_{3}^{i}$. That is to say, $k_{j}^{i}$ denotes the vertex in the i-th column and the j-th row. If $y_{i}^{\left(AorB\right)}=1$, there are four vertices on each column, the specific form of which is shown in Figure 8.

The randomness of the structure prevents the adversary from forging data.

**Step 2: Encrypt data and encode graph state.** Alice and Bob prepare private keys $X_{A}=\left\{x_{i}^{A};i=1,2,\ldots \ldots ,m;x_{i}^{A}\in \left\{0,1\right\}\right\}$ and $X_{B}=\left\{x_{i}^{B};i=1,2,\ldots \ldots ,m;x_{i}^{B}\in \left\{0,1\right\}\right\}$, respectively. Alice and Bob first encrypt the data with X, obtaining secret strings $S_{A}=A⊕X_{A}$; $S_{B}=B⊕X_{B}$. Next, let us consider Alice’s case first, and Bob’s case is similar to Alice’s. Alice determines which bit to encrypt to the data to according to the value of Y. For $y_{i}^{A}=0$, the data are encrypted to $k_{2}^{i}$; for $y_{i}^{A}=1$, the data are encrypted to $k_{3}^{i}$. The encryption method is as follows: For $S_{i}^{A}=0$, no operation is performed; for $S_{i}^{A}=1$, an X or Z gate is randomly applied to the bit to be encrypted. Alice records her encryption method, i.e., records a sequence of I, X, Z (a total of m). I is for $S_{i}^{A}=0$, X or Z is for $S_{i}^{A}=1$. Bob encrypts the data and encodes the graph state in the same way. Alice and Bob send the encoded graph state to the TP through the quantum channel.
**Step 3: Graph state verification and secure summation.** After confirming that all bits have been received, Alice and Bob announce the values of Y to the TP, and the TP verifies and decodes the graph state according to the values of Y. For $y_{i}=0$, the TP removes the stabilizer containing the $k_{2}^{i}$ bit, i.e., only keeps the first row of quantum bits; for $y_{i}=1$, the TP removes the stabilizer containing the $k_{3}^{i}$ bit, i.e., keeps the first and second rows of quantum bits; the TP measures all the remaining stabilizers (about 1.5 m + 2), and if there is a stabilizer with a measurement result of −1, it means that the graph state has been damaged during transmission, and the TP terminates the protocol or notifies the sender to resend. If all the stabilizer measurement results are +1, the TP proceeds to the next step.

**Figure 9:**
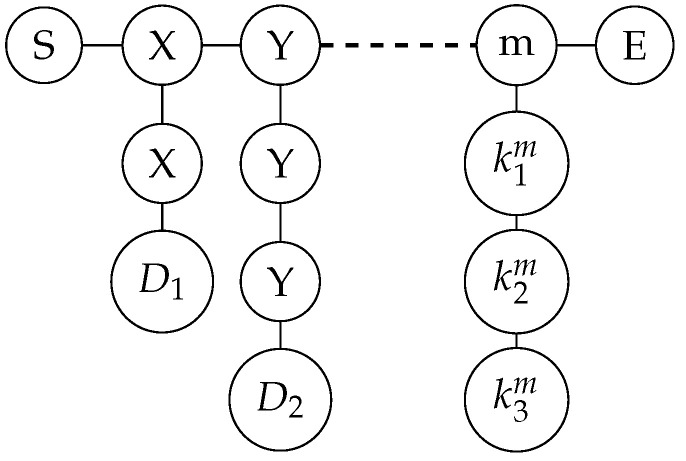
Use different measurement bases for graph states depending on the Y values.

For $y_{i}=0$, the TP performs X-basis measurement on *i*, $k_{1}^{i}$, and keeps $k_{2}^{i}$ as D (data). For $y_{i}=1$, the TP performs Y-basis measurement on *i*, $k_{1}^{i}$, $k_{2}^{i}$, and keeps $k_{3}^{i}$ as D. The graph state is shown in Figure 9.

**Figure 10:**
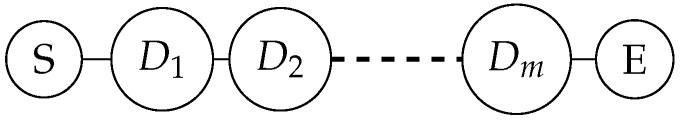
The graph state after measurement and adjustment.

According to the measurement results in the X and Y bases, the graph state is transformed into a new graph state by applying gate operations to adjust the state. The new graph state is shown in Figure 10.

The specific methods of measuring in the X, Y bases and adjusting the graph state are described in Section 2 of this paper. After obtaining the new graph state, the TP performs the inverse operations and measurements on Alice and Bob’s graph states and performs modulo 2 addition on the results, obtaining the secret string D. That is, $D=D_{A}⊕D_{B}$.

**Step 4: Decryption.** Alice and Bob make the decryption keys according to their own encryption sequences (consisting of m gate operations of I, X, and Z), $X_{A}$, and $X_{B}$. The specific method is, for each X gate, apply bit flip to the adjacent three bits in $X_{A}$ and $X_{B}$. For example, if Alice applies an X gate to the i-th bit, then apply bit flip to {$X_{i-1}^{A}$, $X_{i}^{A}$, $X_{i+1}^{A}$}, obtaining the decryption key $X_{A}^{\prime }$; Bob makes the decryption key $X_{B}^{\prime }$ according to his own encryption sequence and $X_{B}$. The final decryption key is obtained by performing modulo 2 addition on $X_{A}^{\prime }$ and $X_{B}^{\prime }$. That is, $X^{\prime }=X_{A}^{\prime }⊕X_{B}^{\prime }$. There are three ways to calculate $X^{\prime }$, one is for Alice to send $X_{A}$ to Bob, and Bob makes $X^{\prime }$ and sends it to the TP. The second is for Alice and Bob to send $X_{A}^{\prime }$ and $X_{B}^{\prime }$ to a semi-honest third party $TP^{\prime }$, and $TP^{\prime }$ sends the calculated $X^{\prime }$ to the TP. The third is to use the property introduced in the first part of Section 2.4 for the TP to send two vertices in a group of graph states to Alice and Bob, respectively. Alice and Bob randomly choose X or Z gates to encode according to their own data, and the TP calculates the modulo 2 sum of Alice and Bob’s decryption keys.

In summary, after the TP obtains $X^{\prime }$, it can decrypt the sum data D, $f\left(A,B\right)=D⊕X^{\prime }$. The protocol ends. f(A,B) is the final summation result.

### 3.2. Protocol 2: Secure Multi-Party Summation Protocol

**Figure 11:**
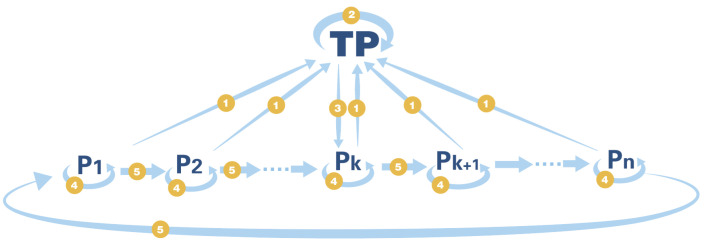
The process of Multi-party summation. ➀: Encoded Graph State. ➁: Secure Summation. ➂: Encrypted Data. ➃: Decrypt Data. ➄: Partially Decrypted Data.

Protocol description: Multiple participants encrypt and sum their respective data with the assistance of a third party (TP). The TP only knows the final summation result, but does not know the specific values of each participant. This protocol is developed based on the secure two-party summation protocol, and has a wide range of application scenarios in the current technology background of cloud computing and big data. Let $P=\{P_{k};k=1,2,\ldots \ldots ,n\}$ be the set of participants, and $P_{k}$ be the k-th participant. Let $P_{k}$’s data be $T_{k}=\left\{t_{i}^{k};i=1,2,\ldots \ldots ,m;t_{i}^{k}\in \left\{0,1\right\}\right\}$, where m is a value far greater than the number of bits of each participant’s data agreed by all participants, and the goal of the protocol is to obtain $f\left(T_{1},T_{2},\ldots \ldots T_{n}\right)=\overset{n}{\underset{k=1}{⊕}}T_{k}$ without leaking $T_{k}$. The secure multi-party summation protocol is shown in Figure 11.

The specific steps of the protocol are as follows:

**Step 1: Prepare the graph state.** Each participant generates a random key $Y_{k}=\{y_{i}^{k};$ $i=1,2,\ldots \ldots ,m;y_{i}^{k}\in \left\{0,1\right\}\}$, and prepares the graph state according to the value of Y. When $y_{i}^{k}=0$, there are three vertices on each column; when $y_{i}^{k}=1$, there are four vertices on each column, the specific form of which is shown in Figure 8.
**Step 2: Encrypt the data and encode the graph state.** Each participant $P_{k}$ prepares a random private key $X_{k}=\left\{x_{i}^{k};i=1,2,\ldots \ldots ,m;x_{i}^{k}\in \left\{0,1\right\}\right\}$, and encrypts the data, obtaining the secret string $S_{k}=T_{k}⊕X_{k}$. According to the value of $S_{k}$, $P_{k}$ prepares the third group of random private keys $Z_{k}=\left\{z_{i}^{k};i=1,2,\ldots \ldots ,m;z_{i}^{k}\in \left\{I,X,Z\right\}\right\}$, where the rule is: when $S_{i}^{k}=0$, $z_{i}^{k}=I$; when $S_{i}^{k}=1$, $z_{i}^{k}=\left\{X,Z\right\}$, that is, randomly choose X or Z gate. At this point, each participant has three groups of random keys, each with its own function: $X_{k}$ is used to encrypt the original data, preventing the TP from obtaining the original data by inference after measurement; $Y_{k}$ is used to randomly select the graph state structure, preventing eavesdroppers on the quantum channel from obtaining the data; $Z_{k}$ is used to encrypt the data with random gate operations, preventing eavesdroppers from stealing the data after the participants disclose the value of $Y_{k}$.

Next, use the graph state to encrypt and encode: $P_{k}$ determines which bit to encode the data to according to the value of $Y_{k}$. For $y_{i}^{k}=0$, encode the data to $k_{2}^{i}$; for $y_{i}^{k}=1$, encode the data to $k_{3}^{i}$. The encryption method is to apply the corresponding gate operation to the bit according to the value of $z_{i}^{k}$ {I,X,Z}. $P_{k}$ sends the encoded graph state to the TP through the quantum channel.

**Step 3: Graph state verification and secure summation.** After confirming that all the bits have been received, all the participants P disclose the value of Y, and the TP verifies and decodes the graph state according to the value of Y. For $y_{i}^{k}=0$, the TP removes the stabilizer containing the $k_{2}^{i}$ bit, that is, only keeps the first row of quantum bits; for $y_{i}^{k}=1$, the TP removes the stabilizer containing the $k_{3}^{i}$ bit, that is, keeps the first and second rows of quantum bits; the TP measures all the remaining stabilizers (about 1.5m+2 for each participant), and if there is a stabilizer with a measurement result of −1, it means that the graph state has been damaged during transmission, and the TP terminates the protocol or notifies the sender to resend. If all the stabilizer measurement results are +1, the TP proceeds to the next step.

For $y_{i}^{k}=0$, the TP performs X-basis measurement on *i*, $k_{1}^{i}$, and keeps $k_{2}^{i}$ as D (data). For $y_{i}^{k}=1$, the TP performs Y-basis measurement on *i*, $k_{1}^{i}$, $k_{2}^{i}$, and keeps $k_{3}^{i}$ as D. The graph state is shown in Figure 9.

According to the measurement results in the X and Y bases, the graph state is transformed into a new graph state by applying gate operations to adjust the state.The new graph state is shown in Figure 10.

After obtaining the new graph state, the TP performs the inverse operations and measurements on each participant’s graph state and applies modulo 2 addition to the results, obtaining the secret string $D=\overset{n}{\underset{k=1}{⊕}}D_{k}$.

**Step 4: Decryption.** Any participant $P_{k}$ makes the decryption key according to $X_{k}$ and $Z_{k}$. The specific method is, if $z_{i}^{k}=X$, apply bit flip to the adjacent three bits {$X_{i-1}^{k}$, $X_{i}^{k}$, $X_{i+1}^{k}$} in $X_{k}$; if $z_{i}^{k}=Z$ or I, do nothing, and obtain the decryption key $X_{k}^{\prime }$.

The TP randomly selects a participant $P_{k}$, and sends the secret string D to him through the classical channel. The participant $P_{k}$ decrypts the data D as $F_{1}=D⊕X_{k}^{\prime }$, and then $P_{k}$ selects the next participant $P_{k+1}$ from the pool of participants to be decrypted. Note that, to prevent the participants from colluding to crack the data of other participants, $P_{k}$ should randomly select $P_{k+1}$ when choosing. $P_{k}$ sends $F_{1}$ to $P_{k+1}$, and $P_{k+1}$ decrypts $F_{1}$ as $F_{2}=F_{1}⊕X_{k+1}^{\prime }$, until the last participant $P_{k-1}$, $P_{k-1}$ decrypts $F_{n-1}$ as $F_{n}=F_{n-1}⊕X_{k-1}^{\prime }=D⊕X_{1}^{\prime }⊕X_{2}^{\prime }⊕\ldots \ldots ⊕X_{n}^{\prime }$.

$P_{k-1}$ announces the final summation result $f\left(T_{1},T_{2},\ldots \ldots T_{n}\right)=F_{n}$. The protocol ends.

### 3.3. An Example of Protocol 2

Suppose there are three participants, $P_{1}$ holds the data 0101010, $P_{2}$ holds the data 0011010, $P_{3}$ holds the data 0110100, and the expected sum result is $f(T_{1},T_{2},T_{3})=0101010⊕0011010⊕0110100=0000100$.

**Step 1: Prepare the graph state.** Each participant generates a random key $Y_{1}=0101101$, $Y_{2}=0101110$, $Y_{3}=0010010$, and prepares the graph state according to the value of Y; when $y_{i}=0$, there are three vertices on each column; when $y_{i}=1$, there are four vertices on each column.
**Step 2: Encrypt the data and encode the graph state.** The three participants randomly generate private keys $X_{1}=0100100$, $X_{2}=0101110$, $X_{3}=0010010$, and encrypt the data, obtaining the secret string $S_{1}=T_{1}⊕X_{1}=0101010⊕0100100=0001110$, $S_{2}=T_{2}⊕X_{2}=0011010⊕0101110=0110100$, $S_{3}=T_{3}⊕X_{3}=0110100⊕0010010=0100110$. According to the value of S, the three participants P make the key Z, $Z_{1}=IIIXXZI$, $Z_{2}=IXXIZII$, $Z_{3}=IXIIZXI$. The three participants determine which bit to encode the data to according to the value of Y. For $y_{i}=0$, the data are encoded to the third quantum bit on the column; for $y_{i}=1$, the data are encoded to the fourth quantum bit on the column. The encryption method is to apply the corresponding gate operation to the bit in the set of $z_{i}$ {I,X,Z}. $P_{1}$ sends the encoded graph state to the TP through the quantum channel.
**Step 3: Graph state verification and secure summation.** After confirming that all bits have been received, all participants P announce the value of Y, and the TP verifies and decodes the graph state according to the value of Y. Taking participant $P_{1}$ as an example, $Y_{1}=0101101$. When $y_{1}=0$, the TP removes the stabilizer containing the bit $k_{2}^{1}$, that is, only keeps the first row of quantum bits; when $y_{2}=1$, the TP removes the stabilizer containing the bit $k_{3}^{2}$, that is, he keeps the first and second rows of quantum bits; the following situations are similar. The final determined stabilizers are shown in Table 3.

The TP performs stabilizer measurement; if there is a stabilizer with measurement result of −1, it means that the graph state has been damaged during transmission, and the TP terminates the protocol or notifies the sender to resend. If all stabilizer measurement results are +1, the TP continues to the next step.

The TP simplifies the graph state; for $y_{i}=0$, the TP applies X basis measurement to *i* and $k_{1}^{i}$, and keeps $k_{2}^{i}$ as $D_{i}$ (data). For $y_{i}=1$, the TP applies Y basis measurement to *i*, $k_{1}^{i}$, and $k_{2}^{i}$, and keeps $k_{3}^{i}$ as $D_{i}$. Afterwards, the TP obtains a 1D linear graph state, the TP applies the inverse operation and measurement to the graph state, and obtains the measurement result. $D_{1}=0011100$, $D_{2}=1111100$, $D_{3}=1010001$, $D=D_{1}⊕D_{2}⊕D_{3}=0110001$.

**Step 4: Decryption.** The three participants calculate the decryption key separately, if $z_{i}=X$, apply bit flip to the adjacent three bits {$X_{i-1}$, $X_{i}$, $X_{i+1}$} in *X*; if $z_{i}=Z$ or I, do nothing, and obtain the decryption key $X_{1}^{\prime }=0110110$, $X_{2}^{\prime }=1100110$, $X_{3}^{\prime }=1100101$. The TP randomly selects a participant $P_{k}$, taking $P_{1}$ as an example, and sends the secret string D to him through the classical channel. The participant $P_{1}$ decrypts the data D as $F^{1}=D⊕X_{1}^{\prime }=0110001⊕0110110=0000111$, $P_{1}$ selects the next participant from the pool to be decrypted, taking $P_{2}$ as an example, $F^{2}=F^{1}⊕X_{2}^{\prime }=0000111⊕1100110=1100001$, the same operation, $F^{3}=F^{2}⊕X_{3}^{\prime }=1100001⊕1100101=0000100$. This is the final sum result, which is consistent with the expected sum result, and the protocol ends.

### 3.4. Adding or Deleting Participants

Based on the secure multi-party summation protocol provided in this paper, participants can be added or deleted arbitrarily. Due to the fact that the TP does not need to prepare quantum states in advance and send them to the relevant participants, how many participants participate in the summation is completely determined by the participants themselves. Before the decryption operation in step 4 is executed (the TP can announce a deadline), all participants can freely choose to participate or quit. The added participants can participate in this round of secure multi-party summation calculation by sending the graph state to the TP and announcing their graph state structure; participants who have participated and announced the graph state structure can also notify the TP that they no longer participate in this round of secure multi-party summation calculation. In addition, if the graph state submitted by some participants fails the stabilizer measurement, then the participant cannot participate in this round of calculation, and the protocol can still be executed normally. After the deadline, the TP no longer accepts new submissions. The TP sums up the data of the participants who passed the stabilizer measurement. And send the sum result to the qualified participants for joint decryption, and to obtain the final sum result.

### 3.5. Protocol 3: A Simplified, Ring-Shaped Secure Multi-Party Summation Protocol without TP

**Figure 12:**
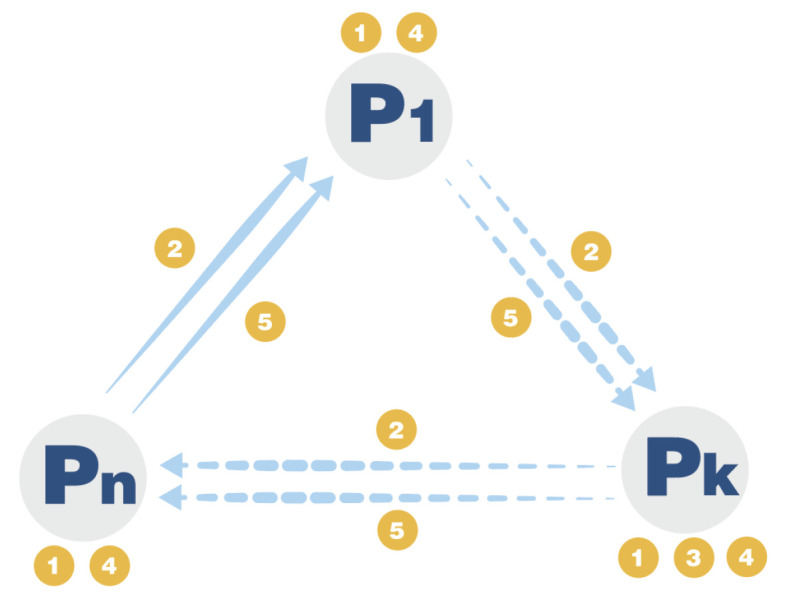
The process of ring-shaped secure multi-party summation protocol without TP. The protocol starts from random participant $P_{k}$. ➀: Encode Graph State. ➁: Encoded Graph State. ➂: Decode Graph State. ➃: Decrypt Data. ➄: Partially Decrypted Data.

Protocol description: Based on protocol 2, we propose a simplified, ring-shaped secure multi-party summation protocol without the help of a semi-honest third party. Let n participants be $P=\{P_{k};k=1,2,\ldots \ldots ,n\}$. Let $P_{k}$ hold the data $T_{k}=\left\{t_{i}^{k};i=1,2,\ldots \ldots ,m;t_{i}^{k}\in \left\{0,1\right\}\right\}$, the goal of the protocol is to obtain $f\left(T_{1},T_{2},\ldots \ldots T_{n}\right)=\overset{n}{\underset{k=1}{⊕}}T_{k}$ without leaking $T_{k}$. The specific protocol is shown in Figure 12.

**Figure 13:**
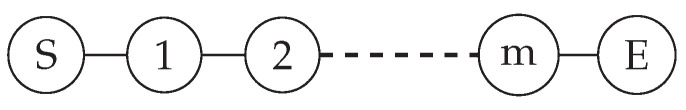
Participant k prepares the initial graph state.

The following are the specific steps of the protocol:

**Step 1: Select the initial participant and prepare the graph state.** The system randomly selects a participant $P_{k}$ as the initial participant, who prepares the initial graph state is shown in Figure 13.
**Step 2: Encrypt data and encode graph state.** Each participant $P_{k}$ prepares a random private key $X_{k}=\left\{x_{i}^{k};i=1,2,\ldots \ldots ,m;x_{i}^{k}\in \left\{0,1\right\}\right\}$, and encrypts the data, obtaining the secret string $S_{k}=T_{k}⊕X_{k}$. According to the value of $S_{k}$, $P_{k}$ prepares a second group of random private keys $Z_{k}=\left\{z_{i}^{k};i=1,2,\ldots \ldots ,m;z_{i}^{k}\in \left\{I,X,Z\right\}\right\}$, where the rule is: when $S_{i}^{k}=0$, $z_{i}^{k}=I$; when $S_{i}^{k}=1$, $z_{i}^{k}=\left\{X,Z\right\}$, that is, randomly choose X or Z gate. At this point, each participant has two groups of random keys, $X_{k}$ is used to encrypt the original data, preventing other participants from measuring and stealing the original data; $Z_{k}$ uses random gate operations to encrypt the data, preventing external attackers from eavesdropping and stealing the data.

Next, use the graph state to encrypt and encode: $P_{k}$ applies the corresponding gate operation to the bit according to the value of $z_{i}^{k}$ {I,X,Z}, noting that the S and E bits are not encoded. $P_{k}$ sends the encoded graph state to the next participant $P_{k+1}$ through the quantum channel, and detects whether the quantum channel is eavesdropped by adding decoy bits, announcing positions, measuring, etc.

**Step 3: Secure summation.** After all participants have encoded their data in turn, they send it back to the initial participant $P_{k}$, who performs the inverse operation and measurement on the jointly encrypted graph state of all participants, obtaining the modulo 2 sum of the secret strings of n participants $D=\overset{n}{\underset{k=1}{⊕}}D_{k}$. The modulo 2 process is completed during the gate operation of n participants on the graph state. The measurement result D is the result after the modulo 2 sum.
**Step 4: Joint decryption.** The initial participant $P_{k}$ makes the decryption key according to encryption sequences $X_{k}$ and $Z_{k}$. The specific method is, if $z_{i}^{k}=X$, apply bit flip to the adjacent three bits {$X_{i-1}^{k}$, $X_{i}^{k}$, $X_{i+1}^{k}$} in $X_{k}$; if $z_{i}^{k}=Z$ or I, do nothing, and obtain the decryption key $X_{k}^{\prime }$. $P_{k}$ decrypts the data D as $F_{1}=D⊕X_{k}^{\prime }$, and then $P_{k}$ randomly selects the next participant $P_{k+1}$ from the pool of participants to be decrypted. $P_{k}$ sends $F_{1}$ to $P_{k+1}$, and $P_{k+1}$ decrypts $F_{1}$ as $F_{2}=F_{1}⊕X_{k+1}^{\prime }$, until the last participant $P_{k-1}$, $P_{k-1}$ decrypts $F_{n-1}$ as $F_{n}=F_{n-1}⊕X_{k-1}^{\prime }=D⊕X_{1}^{\prime }⊕X_{2}^{\prime }⊕\ldots \ldots ⊕X_{n}^{\prime }$.

$P_{k-1}$ announces the final summation result $f\left(T_{1},T_{2},\ldots \ldots T_{n}\right)=F_{n}$. The protocol ends.

## 4. Experimental Verification

To verify the correctness and practicality of the protocol, we conducted experimental verification using IBM’s quantum cloud platform. Protocol 1 and Protocol 3 are simplified versions of Protocol 2. For simplicity, we take Protocol 2 as an example for experimental verification. We will explain step by step how to conduct the experiment according to the steps of Protocol 2. For convenience of presentation, we define the data bit m = 5, the number of participants n = 3, and considering that QCEngine shows quantum circuits more friendly, some key algorithms of quantum circuits are shown by QCEngine.

**Figure 14:**
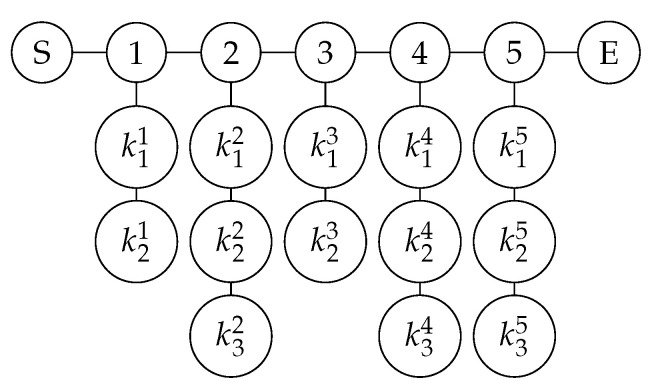
The initial graph state prepared by participant $P_{1}$ according to Y.

**Step 1: Prepare graph state.** Each participant prepares a graph state according to the value of Y. We take $P_{1}$ as an example, whose Y value is $Y_{1}=01011$. When $y_{i}=0$, there are three vertices on each column; when $y_{i}=1$, there are four vertices on each column. The graph state structure prepared according to the Y value is shown in Figure 14.

**Figure 15:**
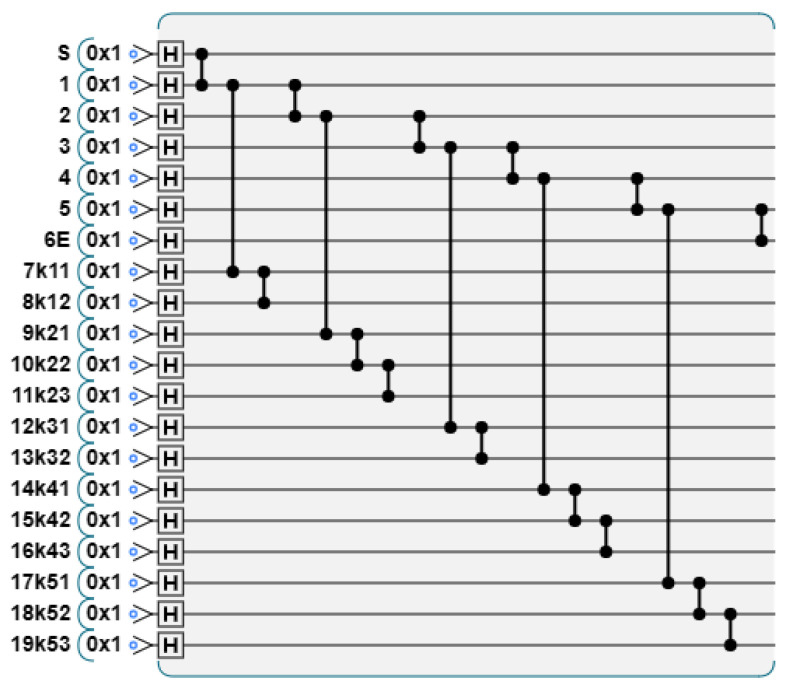
The initial graph state prepared by participant $P_{1}$. Apply H gate to each vertex, and apply CZ gate to the connected vertices.

The generated quantum circuit diagram is shown in Figure 15.

**Figure 16:**
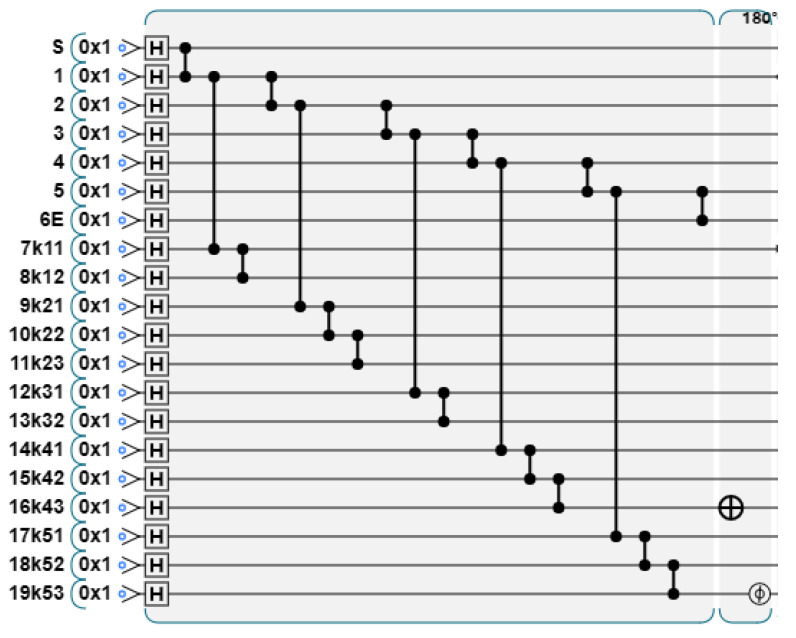
The encoded graph state by participant $P_{1}$. Apply the corresponding gate operation to the bit according to $Z_{1}$. That is, apply X gate to $k_{3}^{4}$, and apply Z gate to $k_{3}^{5}$.

**Step 2: Encode the graph state.** Each participant encodes the graph state according to their own data. We take $P_{1}$ as an example. Its data are $T_{1}=01010$, and the randomly generated private key is $X_{1}=01001$. $S_{1}=T_{1}⊕X_{1}=00011$. According to the value of S, $P_{1}$ made the key $Z_{1}=IIIXZ$, and the quantum circuit diagram after encoding is shown in Figure 16.

For simplicity, the transmission and reception of quantum bits are no longer simulated. It is assumed that the TP has received the graph state submitted by $P_{1}$.

**Step 3: Graph state verification and secure summation.**$P_{1}$ announces the value of Y, and the TP verifies and decodes the graph state according to the value of Y. For $y_{i}=0$, the TP removes the stabilizer containing the bit $k_{2}^{i}$, that is, only keeps the first row of quantum bits; for $y_{i}=1$, the TP removes the stabilizer containing the bit $k_{3}^{i}$, that is, keeps the first and second rows of quantum bits. The Y value of $P_{1}$ is $Y_{1}=01011$, and the stabilizer of the graph state is shown in Table 4.

**Figure 17:**
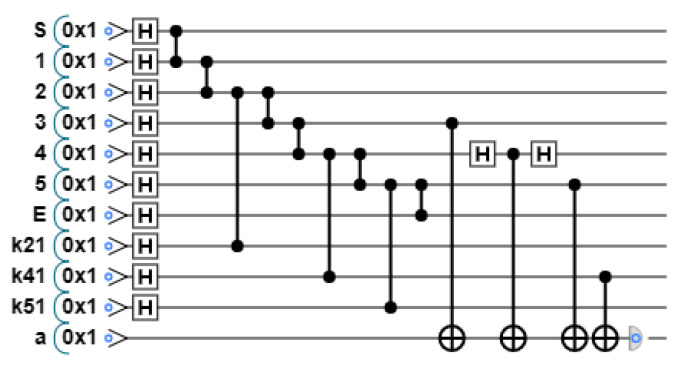
TP performs stabilizer measurement. The stabilizer of vertex 4 is IIIZXZIIZI. For ecah Z, apply CNOT gate; for each X, apply H gate, CNOT gate, H gate. Specifically, apply CNOT(3,a), H(4), CNOT(4,a), H(4), CNOT($k_{1}^{4}$,a). Then, measure the a bit on the Z basis.

We take vertex 4 as an example to perform stabilizer measurement. The quantum circuit diagram after stabilizer measurement is shown in Figure 17.

The TP can confirm whether the quantum graph state has been eavesdropped during transmission by multiple measurements. If the graph state structure remains intact, all stabilizers are in the +1 eigenstate, and the auxiliary bit is consistent with the initial setting value. If the graph state structure changes or some bit is affected by entanglement measurement, measurement retransmission or pauli gate, etc., the auxiliary bit will flip, and the eavesdropping behavior will be detected.

**Figure 18:**
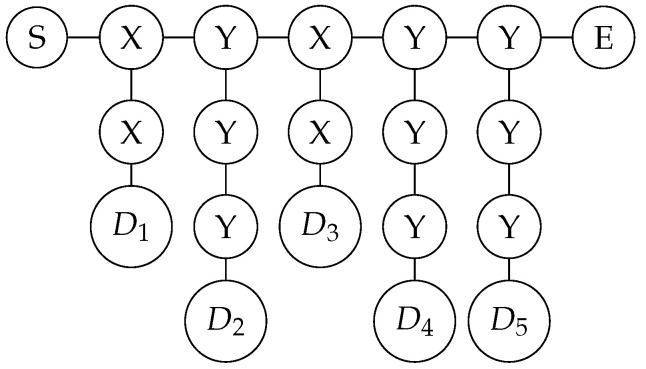
TP uses different measurement bases for graph states depending on the Y values announced by $P_{1}$.

For the graph state that has passed the stabilizer measurement, the TP will simplify it according to the announced graph state structure, and adjust the graph state to a linear shape to facilitate the summation operation. As mentioned earlier, according to the graph state structure announced by $P_{1}$, the TP performs the measurement, and the graph state structure obtained is shown in Figure 18.

**Figure 19:**
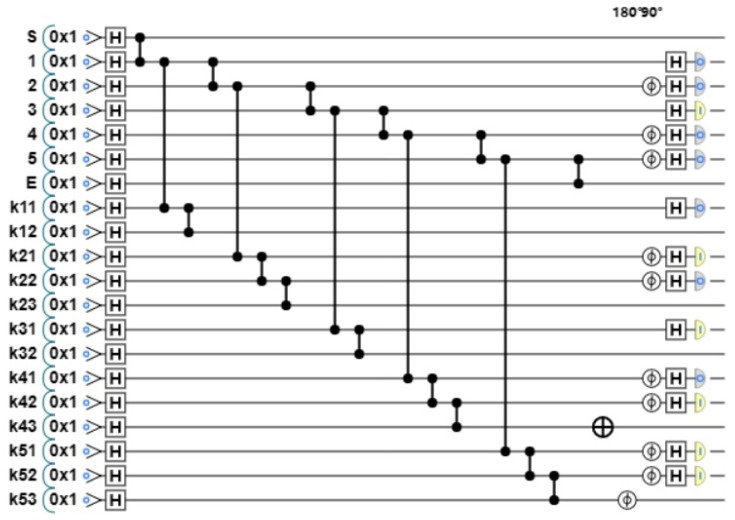
Measurement according to the Y value announced by $P_{1}$. When $y_{i}=0$, apply X-basis measurement to the first two layers, and when $y_{i}=1$, apply Y-basis measurement to the first three layers.

The quantum circuit diagram is shown in Figure 19.

**Figure 20:**
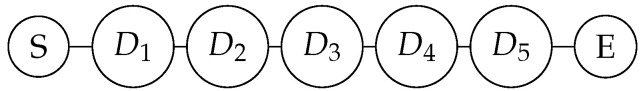
The graph state structure after adjustment.

According to the results of X-basis measurement and Y-basis measurement, the original graph state is adjusted appropriately, and the new graph state structure is shown in Figure 20.

**Figure 21:**
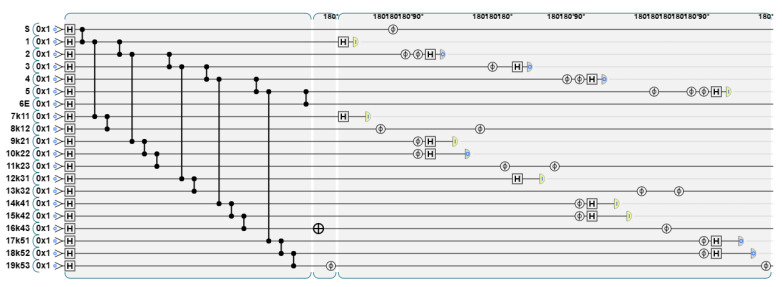
Adjust the graph state according to the measurement results.

The adjustment method according to the measurement results is detailed in Section 2. One of the cases of the quantum circuit diagram is shown in Figure 21. Apply X-basis measurement to vertex 1 and $k_{1}^{1}$, and the measurement result is 11. According to the adjustment rule, apply Z gate to $k_{2}^{1}$, and apply Z gate to S and vertex 2. Then, $k_{2}^{1}$ replaces vertex 1. Apply Y-basis measurement to vertex 2, $k_{1}^{2}$, and $k_{2}^{2}$, and the measurement result is 010. Apply Z gate to $k_{2}^{1}$, vertex 3, and $k_{3}^{2}$. Then, $k_{3}^{2}$ replaces vertex 2. Apply X-basis measurement to vertex 3 and $k_{1}^{3}$, and the measurement result is 01, apply Z gate to $k_{3}^{2}$ and vertex 4; then, $k_{2}^{3}$ replaces vertex 3. Apply Y-basis measurement to vertex 4, $k_{1}^{4}$, and $k_{2}^{4}$, and the measurement result is 011, apply Z gate to $k_{3}^{4}$; then, $k_{3}^{4}$ replaces vertex 4. Apply Y-basis measurement to vertex 5, $k_{1}^{5}$, and $k_{2}^{5}$, and the measurement result is 100, apply Z gate to $k_{3}^{5}$; then, $k_{3}^{5}$ replaces vertex 5. At this point, the original graph state has been adjusted to a linear shape.

**Figure 22:**
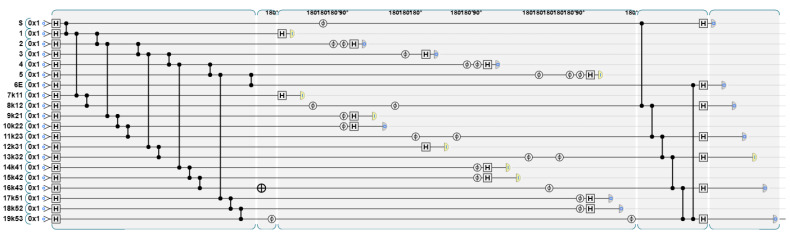
Perform the inverse operation and measurement of the graph state.

**Figure 23:**
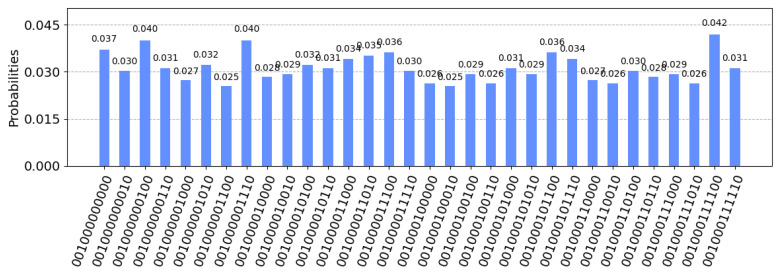
The result of quantum circuit diagram.

The TP applies the inverse operation and measurement of the graph state. The quantum circuit diagram and the result are shown in Figure 22 and Figure 23.

The figure shows the measurement results of the vertices S to E and the newly added vertices D1–D5. For simplicity, we omit the measurement results of other nodes. From the measurement results, it can be seen that the original vertices 1–5 are in the maximum entangled state, and the data-carrying D1–D5 are in the unique eigenstate. The data carried are 0001000, corresponding to the vertices S, D1, D2, D3, D4, D5, E, respectively. In this way, the encrypted data of $P_{1}$ are obtained.

The TP performs the above operations on the graph states submitted by all participants and obtains the measurement results, as shown in Table 5.

The TP sums up the measurement results D=0001000⊕0000001⊕0101010=0100011.

**Step 4: Joint decryption.** Each participant makes a decryption key according to $X_{k}$ and $Z_{k}$. The specific method is, if $z_{i}^{k}=X$, apply bit flip to the adjacent three bits {$X_{i-1}^{k}$, $X_{i}^{k}$, $X_{i+1}^{k}$} in $X_{k}$; if $z_{i}^{k}=Z$ or I, do nothing, and obtain the decryption key $X_{k}^{\prime }$. For this example, $X_{1}^{\prime }=0011100$, $X_{2}^{\prime }=0001101$, $X_{3}^{\prime }=0110000$. The TP randomly selects a participant $P_{k}$, and sends the secret string D to him through the classical channel. In this example, it is assumed that the three participants decrypt in turn. $F^{1}=D⊕X_{1}^{\prime }=0100011⊕0011100=0111111$, $F^{2}=F^{1}⊕X_{2}^{\prime }=0111111⊕0001101=0110010$, $F^{3}=F^{2}⊕X_{3}^{\prime }=0110010⊕0110000=0000010$, $F^{3}=f\left(T_{1},T_{2},T_{3}\right)$ is the final summation result, and the protocol ends.

**Verification:** The original data sum is $T_{1}⊕T_{2}⊕T_{3}=01010⊕00110⊕01101=00001$, $F^{3}$ removes the head and tail vertices S and E, which is consistent with the expected result, further confirming the correctness and effectiveness of the protocol.

## 5. Protocol Analysis

Next, we analyze the protocols we provide, including correctness analysis, security analysis, and comparative analysis.

### 5.1. Correctness Analysis

Secure two-party summation is a special case of secure multi-party summation, so this section mainly focuses on the correctness analysis of secure multi-party summation.

In the secure multi-party summation protocol, the final summation result we obtain is $f\left(T_{1},T_{2},\ldots \ldots T_{n}\right)=F^{n}$, and we need to verify that $F^{n}=\overset{n}{\underset{k=1}{⊕}}T_{k}$. We proceed with the theoretical derivation. We define an operation $U\left(H,J\right);H_{i}\in \left\{I,X,Z\right\};J_{i}\in \left\{0,1\right\}$, which operates on $J_{i}$ according to the information of $H_{i}$. When $H_{i}=X$, it performs bit flip on the three bits $J_{i-1}$, $J_{i}$, and $J_{i+1}$. It is easy to see that $U^{2}=I$.

Each participant’s original data are $T_{k}$, and in step 2, they first participant encrypt the data with X, obtaining secret strings $S_{k}=T_{k}⊕X_{k}$. According to the encryption of the graph state structure by Y, it is transformed into the same structure after the TP decrypts it, which can be ignored in the correctness analysis. According to $S_{k}$, each participant randomly generates $Z_{k}$, and then encodes the graph state and sends it to the TP through the quantum channel. The TP then performs the inverse operation and measurement of the graph state and recovers the data. As shown in the properties of the graph state in Section 2, this process is actually equivalent to applying $U(Z_{k},S_{k})$. The process of performing bit flip on a bit Q is actually the process of Q⊕1, which means that applying $U(Z_{k},S_{k})$ is actually equivalent to performing modulo 2 addition of a string of 0s and 1s with $S_{k}$, that is, $U\left(Z_{k},S_{k}\right)=U\left(Z_{k},\left\{0,0,......,0\right\}\right)⊕S_{k}$. Therefore, $U\left(Z_{k},S_{k}\right)=U\left(Z_{k},T_{k}⊕X_{k}\right)=T_{k}⊕U\left(Z_{k},X_{k}\right)$.

In step 3, the TP obtains the secret string $D=\overset{n}{\underset{k=1}{⊕}}D_{k}=\overset{n}{\underset{k=1}{⊕}}U\left(Z_{k},S_{k}\right)=\overset{n}{\underset{k=1}{⊕}}U\left(Z_{k},T_{k}⊕X_{k}\right)=\overset{n}{\underset{k=1}{⊕}}\left(T_{k}⊕U\left(Z_{k},X_{k}\right)\right)$.

In step 4, each participant makes the decryption key $U(Z_{k},X_{k})$ and decrypts it. Due to the fact that $U^{2}\left(Z_{k},X_{k}\right)=I$, $F^{1}=D⊕X_{k}^{\prime }=D⊕U\left(Z_{k},X_{k}\right)=\left(\overset{n}{\underset{j=1}{⊕}}T_{j}\right)⊕\left(\overset{k-1}{\underset{j=1}{⊕}}U\left(Z_{j},X_{j}\right)\right)⊕\left(\overset{n}{\underset{j=k+1}{⊕}}U\left(Z_{j},X_{j}\right)\right)$. After each participant decrypts, the result is $F^{n}=f\left(T_{1},T_{2},\ldots \ldots T_{n}\right)=\overset{n}{\underset{k=1}{⊕}}T_{k}$. The protocol ends.

### 5.2. Security Analysis

The security guarantee of the secure multi-party computation protocol based on graph state mainly relies on the randomness of the graph state structure and the randomness of the encryption gate operation. Next, we will analyze the external attack and internal attack separately. Considering that the secure two-party summation protocol is a special case of the secure multi-party summation protocol, we mainly analyze the secure multi-party summation protocol.

#### 5.2.1. External Attack

External attack mainly refers to the attack on the quantum channel by eavesdroppers, who can perform entanglement measurement, intercept–resend, and measure–resend attacks. This protocol can ensure that the original data are not leaked in the presence of external attackers.

**Theorem** **1.** *It is impossible to restore the information carried by the graph state without knowing the graph state structure.*

**Proof of Theorem 1.**  Take the simplest 1D two-vertex graph state shown in Figure 2 as an example. Its initial state is $\frac{1}{2}\left(|00\rangle +|01\rangle +|10\rangle -|11\rangle \right)$, which is in the maximally entangled state. We apply X gate, Z gate, or I gate randomly to the two qubits, resulting in nine possible outcomes, which are II, IX, IZ, XI, XX, XZ, ZI, ZX, and ZZ. Among them, II is consistent with the original state, and the other eight states will cause the initial state to change, but the result is still in the maximally entangled state. The specific changes are shown in Table 6.

**Table 6:** Table of graph state changes.

Gate Operation	Quantum State
II	$\frac{1}{2}\left(|00\rangle +|01\rangle +|10\rangle -|11\rangle \right)$
IX	$\frac{1}{2}\left(|01\rangle +|00\rangle +|11\rangle -|10\rangle \right)$
IZ	$\frac{1}{2}\left(|00\rangle -|01\rangle +|10\rangle +|11\rangle \right)$
XI	$\frac{1}{2}\left(|10\rangle +|11\rangle +|00\rangle -|01\rangle \right)$
XX	$\frac{1}{2}\left(|11\rangle +|10\rangle +|01\rangle -|00\rangle \right)$
XZ	$\frac{1}{2}\left(|10\rangle -|11\rangle +|00\rangle +|01\rangle \right)$
ZI	$\frac{1}{2}\left(|00\rangle +|01\rangle -|10\rangle +|11\rangle \right)$
ZX	$\frac{1}{2}\left(|01\rangle +|00\rangle -|11\rangle +|10\rangle \right)$
ZZ	$\frac{1}{2}\left(|00\rangle -|01\rangle -|10\rangle +|11\rangle \right)$

For quantum bits in the maximally entangled state, the probability of obtaining each state is completely equal, and the original encryption information cannot be obtained by measurement. Other graph states have been introduced in detail in Section 2, and they are all in the maximally entangled state after protocol encoding. The eavesdropper EVE or the receiver TP cannot restore the original information without knowing the graph state structure. Therefore, this encryption method is a perfect quantum encryption method, which meets the requirements of information-theoretic security. □

Next, we introduce several attack scenarios that are commonly encountered in protocol applications:

**Scenario 1: An external attacker intercepts the information of a participant and tries to obtain the original data.**

**Analysis:** In step 1 and step 2, all participants P randomly prepared the graph state structure and encoded the graph state according to the random key. In step 2, EVE obtained the quantum bits sent by a participant $P_{l}$ to TP. In step 3, the TP did not receive all the quantum bits from the predetermined participants, the protocol was terminated, and the participants no longer announced the graph state structure. The eavesdropper EVE obtained about 3.5 m + 2 (m is the number of data bits) quantum bits, but he did not know the original graph state structure and could not determine which quantum bits were the information-bearing quantum bits. In fact, there are m quantum bits that carry information, and these m quantum bits are entangled with other m bits, and the other m bits are entangled with other bits too, forming a complex graph state. If the initial graph state cannot be restored, the eavesdropper’s measurement can only obtain completely random results rather than any useful information.

**Scenario 2: An external attacker tries to create fake data to confuse the sum result.**

**Analysis:** Some external attackers just want to disrupt the operation of the protocol and do not want to steal information. They create fake data and submit them to destroy the sum result. In step 1 and step 2, all participants P randomly prepared the graph state structure and encoded the graph state according to the random key. The external attacker EVE also made and encoded his own graph state structure. Then, there are two situations, one is that EVE directly submits his own quantum bits to the TP, the TP finds that the number of participants is wrong, terminates the protocol, and the sum operation is not performed. The participants can replace the quantum channel with a more secure one. Another situation is that EVE intercepts the data of one of the participants $P_{l}$ and submits his own data. Before the TP confirms that the data of all participants have been received, the participants will not announce the graph state structure, so the graph state structure that the eavesdropper EVE sent to the TP is randomly determined by EVE. Due to the fact that there are $2^{m}$ graph state structures for m information bits, when the information bits are sufficient, the probability that the graph state structure randomly selected by the eavesdropper EVE is consistent with the stolen graph state structure is extremely low. If the number of information bits is small, security can be improved by supplementing the data. In step 3, after the TP confirms that the quantum graph states sent by all participants have been received, the participants announce their graph state structure through the classical channel. The information transmission of the classical channel can be prevented from being faked by external attackers by permission authentication. The TP performs stabilizer measurement according to the graph state structure announced by each participant. Due to the fact that the quantum graph state submitted by EVE is inconsistent with the graph state structure announced by $P_{l}$, it cannot pass the stabilizer measurement, and the TP finds the eavesdropper and terminates the protocol. At the same time, the TP can find out which participant was eavesdropped on. Furthermore, if EVE can both send quantum graph states to the TP through the quantum channel and announce the graph state structure through the classical channel through permission authentication, he will become an internal participant, and internal attacks will be discussed in the next section.

**Scenario 3: An external attacker understands the basic operation of the protocol and tries to intercept the information of a participant.**

**Analysis:** Based on scenario 2, the eavesdropper EVE only wants to obtain the information of a participant $P_{l}$, he does not care whether the protocol will be terminated, nor does he care whether there is an eavesdropper being discovered. According to scenario 2, we know that EVE intercepted $P_{l}^{\prime }s$ quantum graph state in step 2 and submitted his own forged graph state. After the TP announced that the information of all participants had been received, the participants announced their graph state structure, and EVE also stole the graph state structure announced by $P_{l}$ on the classical channel. Due to the fact that the graph state structure he submitted cannot pass the stabilizer measurement, the eavesdropping behavior will be detected as explained in situation 2. In this situation, the eavesdropper EVE only wants to know the original data of $P_{l}$. According to the stolen graph state structure, EVE performed the inverse operation of the graph state and measured it, obtaining the data D. But as we know from the previous text, D is the result of encoding according to the random X set and Z set, and this two sets are not disclosed in the whole process of protocol execution. Therefore, EVE cannot obtain the original data of $P_{l}$.

In summary, for external attackers, they can neither obtain the original data of the participants nor influence the summation result by creating fake data.

#### 5.2.2. Internal Attack

Internal attack is a major threat to protocol security, as it has an advantage in stealing data by knowing the whole mechanism of protocol execution. For any protocol, it can only prevent internal participants from stealing other people’s information, but cannot prevent internal participants from submitting fake data, because their original data can be faked. However, this also shows that, for any protocol, authentication is very necessary, because if an attacker can obtain the authority of an internal participant, he can influence the summation result by submitting fake data. The protocol provided in this paper can resist internal attackers, which means preventing internal attackers from stealing data alone or in collusion with others. It includes the following situations:

**Situation 1: A participant directly intercepts the quantum graph state of other participants, trying to steal the original data of other participants.**

**Analysis:** In this situation, the internal participant is regarded as an external participant, because the graph state structure he submitted cannot pass the stabilizer measurement, and his stealing behavior will be detected. Due to the fact that the stolen participant used random X sets and Z sets to encode the graph state, and prepared the graph state structure with random Y sets, the thief cannot recover the original data, which is the same as the external attacker.

**Situation 2: Two or more participants collude in an attempt to steal the original data from other participants.**

**Analysis:** In step 1, each participant prepares the graph state by themselves. In step 2, each participant encodes the graph state according to the held data and submits it to the TP. In this process, stealing information will be regarded as an external attacker, which has been explained in the previous section. In step 3, the TP performs inverse operations and measurements on the graph state and applies modulo 2 addition to the results, obtaining the summation result. In the first three steps of the execution process, each participant interacts with the TP separately, and does not involve other participants, so the collusion cannot steal the information of other participants. The colluders can only hope to steal the information of other participants in step 4’s decryption process. In step 4, the TP randomly selects a participant as the initial decryptor, who decrypts the data and then randomly selects another participant from the decryption pool. The randomness of the process can ensure that the colluders cannot accurately pinch a participant and steal the data.

Pinching a participant means that two colluding participants $P_{l}$ and $P_{l}+2$ try to obtain the original data of $P_{l}+1$. In this protocol, even if the colluding participants happen to be in the pinching position, they can only obtain the decryption key of the pinched participant. As we can see from step 4 and the correctness analysis, the decryption key is $X_{k}^{\prime }=U\left(Z_{k},X_{k}\right)$, where $U(Z_{k},X_{k})$ is the result of encoding according to the random X set and Z set, and does not carry any information of the original data. Therefore, the colluding participants cannot obtain the original data of other participants by pinching. Even if n-2 participants collude, they cannot obtain the original data of the other two participants. And the secure multi-party summation protocol is based on the premise that n-1 participants do not collude, because n-1 participants can easily deduce the original data of another participant in advance by knowing the summation information.

Here is a simple example to illustrate how internal attacks are prevented. We consider the example in Section 3.3, where $P_{1}$’s private data are 0101010, $P_{2}$’s private data are 0011010, and $P_{3}$’s private data are 0110100. The graph state structure is used to ensure the security of the transmission channel between the participants and the TP, and the focus is on detecting and preventing external attacks, which we do not consider for now. In step 2, the three participants each generate a random key, $X_{1}$ = 0100100, $X_{2}$ = 0101110, $X_{3}$ = 0010010, use it to encrypt the data, and obtain $S_{1}$ = 0001110, $S_{2}$ = 0110100, $S_{3}$ = 0100110. According to $S_{1}$, they generate random gate operations, $Z_{1}$ = IIIXXZI, $Z_{2}$ = IXXIZII, $Z_{3}$ = IXIIZXI. In step 3, the TP performs measurements on the graph state and obtains $D_{1}$ = 0011100, $D_{2}$ = 1111100, $D_{3}$ = 1010001, *D* = $D_{1}⊕D_{2}⊕D_{3}$ = 0110001. Due to the fact that n-1 participants can collude to steal data by simple subtraction, we need to introduce a new participant. $P_{4}$’s private data are 0110010, $X_{4}$ = 0011010, $S_{4}$ = 0101000, $Z_{4}$ = IZIXIII, $D_{4}$ = 0110100, *D* = $D_{1}⊕D_{2}⊕D_{3}⊕D_{4}$ = 0000101. Suppose $P_{1}$ and $P_{2}$ want to obtain $P_{3}$’s data by colluding; due to the randomness of the decryption order, $P_{1}$ and $P_{2}$ have only a 1/3 probability of pinching $P_{3}$. We consider the case of pinching, such as the decryption order is $P_{1}$ $P_{3}$ $P_{2}$ $P_{4}$. In step 4, the four participants each make a decryption key $X_{1}^{\prime }$ = 0110110, $X_{2}^{\prime }$ = 1100110, $X_{3}^{\prime }$ = 1100101, $X_{4}^{\prime }$ = 0000110. After $P_{1}$ decrypts, he obtains the data $D⊕X_{1}^{\prime }$ = 0110011; after $P_{3}$ decrypts, he obtains the data $D⊕X_{1}^{\prime }⊕X_{3}^{\prime }$ = 1010110; after $P_{2}$ decrypts, he obtains the data $D⊕X_{1}^{\prime }⊕X_{3}^{\prime }⊕X_{2}^{\prime }$ = 0110000. At this point, $P_{1}$ and $P_{2}$ want to steal $P_{3}$’s data by colluding, they share the data and perform modulo 2 subtraction (equivalent to addition), the result is 0110011 ⊕ 1010110 = 1100101. Note that they obtain $P_{3}$’s decryption key, but they cannot obtain $P_{3}$’s private data 0110100. Due to the existence of $P_{4}$, they also cannot obtain the original data of another participant by sharing their own private data, so the protocol achieves the security guarantee that even n-2 participants colluding cannot obtain the original data.

**Situation 3: TP tries to steal the original information of the participants.**

**Analysis:** In step 2, each participant sends their encoded graph state to the TP through the quantum channel. In step 3, after the TP confirms that it has received the graph states of all participants, each participant announces their graph state structure, and the TP performs inverse operations and obtains the encoded information $D_{k}$ of each participant, but the TP cannot deduce the original data of participant $P_{k}$ from $D_{k}$. As we can see from the correctness analysis section, $D_{k}=U\left(Z_{k},S_{k}\right)=U\left(Z_{k},X_{k}\right)⊕T_{k}$, where $U(Z_{k},X_{k})$ is the result of encoding according to the random X set and Z set, and the X set, Z set and the original data are completely linearly independent. In the whole process of the protocol execution, $P_{k}$ did not disclose their own X set and Z set, so the TP cannot deduce the original data of participant $P_{k}$ from $D_{k}$.

**Situation 4: TP colludes with some participants to obtain the information of other participants.**

**Analysis:** In step 2, each participant sends their encoded graph state to the TP through the quantum channel. In step 3, after the TP confirms that it has received the graph states of all participants, each participant announces their graph state structure, and the TP performs inverse operations and obtains the encoded information $D_{k}$ of each participant, and the TP sums up the information of each participant to obtain D. In step 4, the TP randomly selects a participant as the initial decryptor, who decrypts the data, and randomly selects another participant from the decryption pool. Theoretically, the randomness can prevent the colluding participants from pinching a certain participant successfully, but if there are too many colluding participants, the probability of pinching a certain participant successfully will increase greatly. The decryption key of the pinched participant will be leaked, and through the collusion of the pinching participant and the TP, the original data of the pinched participant can be obtained. This is also a defect of the tree-structured secure multi-party summation protocol. To address this defect, a ring-shaped secure multi-party summation protocol can be adopted, such as the one proposed in Protocol 3, which is “a simplified, TP-free ring-shaped secure multi-party summation protocol”. In this protocol, the initial participant plays the role of the TP, but it is unaware of the encryption information of each participant and only knows the final summation result (encrypted). Therefore, it cannot recover the original data by colluding with and obtaining the decryption key of any participant. But the ring-shaped secure multi-party summation protocol has higher requirements for real-time performance, and each participant needs to judge whether the quantum communication with other participants is secure, which has a large communication overhead, so it is not suitable for large-scale secure multi-party summation. The semi-honest TP designed in this protocol does not need human intervention, and can be implemented by a third-party program trusted by all participants. With the help of the random decryption order mechanism, it can resist collusion attacks.

### 5.3. Comparative Analysis

Existing QSMS protocols are generally based on single Photons [40,42], entanglement swapping [39], and QFT [38] techniques, which utilize the inherent randomness of quantum physics. The protocol proposed in this paper introduces structural randomness and gate operation randomness on top of the quantum randomness, which further ensures the security of the protocol. Existing QSMS protocols use decoy bits to test the security of the quantum channel, while the protocol 2 provided in this paper uses the stabilizer measurement property of quantum graph states to verify the transmission security, without the need of extra decoy bits, making it more usable. Dou et al. [51] provided a QSMC protocol based on quantum graph states, which employs fixed gate operations to achieve summation, which is easy to break. The protocol in this paper uses techniques such as random graph state structure, random gate operations, and stabilizer measurements, which have more advantages in terms of security and usability. Existing QSMS protocols [37,46] usually fix the number of participants in advance, for the convenience of transmission and decryption. The Protocol 2 proposed in this paper allows for the flexible addition and deletion of participants before the TP completes the computation, as its encryption and computation processes are all performed separately with the TP.

## 6. Conclusions and Discussion

This paper proposes a new quantum secure multi-computation protocol based on graph state, which uses the special properties of graph state to ensure the security of data. This paper designs three encryption protocols, all of which are used to solve the classic problems in the field of secure multi-party computation. The protocols are based on random graph state structures, random gate operations, and random encryption keys, providing higher security performance for data security. This paper provides experimental verification, correctness analysis, and security analysis, fully demonstrating the practicality, correctness, and security of the protocol. Of course, there are also some shortcomings. First, Protocol 2 requires a significant amount of quantum bits to ensure the security of the graph state structure, which has a large computational overhead and communication overhead. Protocol 3 requires fewer quantum bits, but cannot use stabilizer measurement to verify the channel security, and still needs to use the traditional method of adding decoy bits. Protocol 3 also has higher requirements for real-time performance and is not suitable for large-scale computation. Second, applying graph state to transmit data requires high communication quality of the quantum channel, and the noise of the quantum channel will affect the transmission of the graph state, which requires the use of error correction codes and other methods to improve the practicality of the protocol. In future research, we can consider using the basic properties of the current graph state to solve other secure multi-party computation problems; we can further study the properties of graph state stabilizers and apply the protocol in noisy channels; we can consider further expanding other graph state structures, studying other graph state properties, and applying them to quantum secure multi-party computation; We can further study the integration of graph state with classical encryption methods and other quantum methods, such as entanglement, gate operation, and QFT, to enhance the protocol security.

## Figures and Tables

**Table 1:** The stabilizer of the four-vertex 2D square graph state.

Vertex Number	Gate Operation
1	$X_{1}Z_{2}II$
2	$Z_{1}X_{2}Z_{3}Z_{4}$ ^1^
3	$IZ_{2}X_{3}I$
4	$IZ_{2}IX_{4}$

^1^ For vertex 2, in addition to being connected to vertices 1 and 3 in the horizontal direction, it is also connected to vertex 4 in the vertical direction.

**Table 2:** Truth table of Y-basis measurement for five-vertex star graph state.

Gate Operation	Measurement Result	
*I*	000	111
$Z_{S}Z_{E}$	001	110
$Z_{D}$	100	011
$Z_{S}Z_{E}Z_{D}$	010	101

**Table 3:** Graph state stabilizer of $P_{1}$.

Vertex	Stabilizer
S	XZIIIIIIIIIII
1	ZXZIIIIIIIIII
2	IZXZIIIZIIIII
3	IIZXZIIIIIIII
4	IIIZXZIIZIIII
5	IIIIZXZIIZIII
6	IIIIIZXZIIIII
7	IIIIIIZXZIIZI
E	IIIIIIIZXIIII
$k_{1}^{2}$	IIZIIIIIIXIII
$k_{1}^{4}$	IIIIZIIIXIXII
$k_{1}^{5}$	IIIIIZIIIIIXI
$k_{1}^{7}$	IIIIIIIZIIIIX

**Table 4:** Graph state stabilizer of $P_{1}$.

Vertex	Stabilizer
S	XZIIIIIIII
1	ZXZIIIIIII
2	IZXZIIIZII
3	IIZXZIIIII
4	IIIZXZIIZI
5	IIIIZXZIIZ
E	IIIIIZXIII
$k_{1}^{2}$	IIZIIIIXII
$k_{1}^{4}$	IIIIIZIIXI
$k_{1}^{5}$	IIIIIIZIIX

**Table 5:** The result of measurement.

Participant	Original Data T	Random Private Key X	Secret String S=T⊕X	Random Gate Operation Z	Measurement Result D
$P_{1}$	01010	01001	00011	IIIXZ	0001000
$P_{2}$	00110	01011	01101	IZXIX	0000001
$P_{3}$	01101	00100	01001	IXIIZ	0101010

## Abbreviations

The following abbreviations are used in this manuscript:

QSMS	Quantum secure multi-party summation
QSMC	Quantum secure multi-party computation
TP	Third party
EVE	Eavesdropper
A	Original data of Alice
B	Original data of Bob
$P_{k}$	Participant k
$T_{k}$	Original data of participant k
$X_{k}$	Random private key of participant k
$S_{k}$	Secret string of participant k
$Y_{k}$	Random graph state structure of participant k
$k_{j}^{i}$	The vertex in the i-th column and the j-th row
$Z_{k}$	Random gate operation of participant k
$D_{k}$	Measurement result of participant k
$X_{k}^{\prime }$	Decryption key of participant k
$F^{n}$	Final result
U(H,J)	Operation on J according to the information of H
$H_{i}$	The i-th bit of H
$J_{i}$	The i-th bit of J
